# Functional Prediction and Assignment of *Methanobrevibacter ruminantium* M1 Operome Using a Combined Bioinformatics Approach

**DOI:** 10.3389/fgene.2020.593990

**Published:** 2020-12-16

**Authors:** M. Bharathi, N. Senthil Kumar, P. Chellapandi

**Affiliations:** ^1^Molecular Systems Engineering Lab, Department of Bioinformatics, School of Life Sciences, Bharathidasan University, Tiruchirappalli, India; ^2^Human Genetics Lab, Department of Biotechnology, School of Life Sciences, Mizoram University (Central University), Aizawl, India

**Keywords:** methanobrevibacter, methane mitigation, hypothetical proteins, protein function, molecular machinery

## Abstract

*Methanobrevibacter ruminantium* M1 (MRU) is a rod-shaped rumen methanogen with the ability to use H_2_ and CO_2_, and formate as substrates for methane formation in the ruminants. Enteric methane emitted from this organism can also be influential to the loss of dietary energy in ruminants and humans. To date, there is no successful technology to reduce methane due to a lack of knowledge on its molecular machinery and 73% conserved hypothetical proteins (HPs; operome) whose functions are still not ascertained perceptively. To address this issue, we have predicted and assigned a precise function to HPs and categorize them as metabolic enzymes, binding proteins, and transport proteins using a combined bioinformatics approach. The results of our study show that 257 (34%) HPs have well-defined functions and contributed essential roles in its growth physiology and host adaptation. The genome-neighborhood analysis identified 6 operon-like clusters such as *hsp*, TRAM, *dsr*, *cbs* and *cas*, which are responsible for protein folding, sudden heat-shock, host defense, and protection against the toxicities in the rumen. The functions predicted from MRU operome comprised of 96 metabolic enzymes with 17 metabolic subsystems, 31 transcriptional regulators, 23 transport, and 11 binding proteins. Functional annotation of its operome is thus more imperative to unravel the molecular and cellular machinery at the systems-level. The functional assignment of its operome would advance strategies to develop new anti-methanogenic targets to mitigate methane production. Hence, our approach provides new insight into the understanding of its growth physiology and lifestyle in the ruminants and also to reduce anthropogenic greenhouse gas emissions worldwide.

## Introduction

Enteric methane emission from ruminants is of great concern not only for its impact on global warming potential but also for ensuring the long-term sustainability of ruminant-based agriculture. Methane emission from rumen methanogens (163.3 million metric tons of CO_2_ equivalents) represents a loss of about 5–7% of dietary energy in ruminants (Hristov et al., 2013; Chellapandi et al., 2017a, 2018; Chellapandi and Prathiviraj, 2020). Methanobrevibacter genus is a dominant rumen methanogenic archaea (61.6%) in which *Methanobrevibacter ruminantium* M1 (MRU) accounted for 27.3% (Janssen and Kirs, 2008). MRU is a hydrogenotrophic rumen methanogen that use H_2_ to reduce CO_2_ for methane biosynthesis. It also uses formate as a carbon source for its growth and energy metabolism (Kaster et al., 2011). This is the first genome sequence to be completed for rumen methanogen. It is a circular chromosome (2.93 Mbp) consisting of 2,278 coding-genes and 144 metabolic pathways with 722 reactions, 557 enzymes, and 751 metabolites (Leahy et al., 2010). However, the MRU genome consists of 756 coding-genes (73%) annotated as hypothetical proteins (HPs). It suggests that the entire proteome functions of this organism are not yet known and have to be elucidated to date.

The function of only 50–70% of coding-genes has been annotated with reasonable confidence in the most completely sequenced bacterial genomes using automated genome sequence analysis (Loewenstein et al., 2009). The characterization of proteins with unknown biological function is known as operome (Greenbaum et al., 2001; Chellapandi et al., 2017b; Prathiviraj and Chellapandi, 2019). Putative genes with known orthologs and no orthologs are termed as conserved hypothetical proteins and uncharacterized proteins, respectively (Mazandu and Mulder, 2012; Shahbaaz et al., 2013). Several approaches have been developed for assisting the function of operome from prokaryotic genomes using the information derived from sequence and structural motifs (Sivashankari and Shanmughavel, 2006; Chellapandi et al., 2017b; Singh and Singh, 2018; Prathiviraj and Chellapandi, 2020a; Sangavai et al., 2020). No one has been employed a combined bioinformatics prediction approach including sequence, structure, and literature confidences for functional assignment of operome and its contribution to metabolic subsystems and cellular machinery. A precise annotation of the operome of a particular genome leads to the discovery of new functions for the development of veterinary and human therapeutics (Ijaq et al., 2015).

The conserved domain-based functional assignment was done for HPs from *Pongo abeli*i and *Sus scrofa*. It has provided a hint for genome-wide annotation in poorly understood genomes (Jitendra et al., 2011). The structure-based approach has been applied to predict the function of operome from *Mycoplasma hyopneumoniae* (da Fonsêca et al., 2012). Functional and structural domain analysis (Namboori et al., 2004), integrated genomic context analysis (Yellaboina et al., 2007) and literature mining (Doerks et al., 2012), functional enrichment analysis (Mazandu and Mulder, 2012), and genome-scale fold-recognition (Mao et al., 2013) have been used to annotate the potential function of operome from *Mycobacterium tuberculosis* H37Rv. Sequence-based and structure-based approaches have been used to define and prioritize some HPs from *Candida dubliniensis*, *Vibrio cholerae* O139, and *Staphylococcus aureus* as therapeutic targets for the treatment of their infections in humans (McAdow et al., 2011, 2012; Bharat Siva Varma et al., 2015; Islam et al., 2015). Besides, only one HP (MJ_0577) was functionally annotated in *Methanococcus jannaschii* using a structural-based approach (Zarembinski et al., 1998).

Many *in silico* attempts have been focused on the functional prediction of operome from human pathogens and no reports on rumen methanogens. Several genome-scale metabolic networks have been reconstructed for methanogenic archaea with a low fraction of HPs functionally assigned by sequence similarity analysis (Chellapandi et al., 2018; Prathiviraj and Chellapandi, 2020a). Since, functional annotation of operome is a great concern not only for implementing our fragmentary knowledge on the potential drug targets but also for genome refinement and improved microbial genome-scale reconstructions (Poulsen et al., 2010; Mazandu and Mulder, 2012; Prathiviraj and Chellapandi, 2019). Thus, we have employed a combined bioinformatics approach for functional assignment, and categorization of operome from MRU with a biological knowledgebase. The predicted functions of operome allow us to comprehend its growth physiology and metabolic behavior in the rumen environment. Several methanogenic antibiotics, inhibitors, and vaccines have been currently available for enteric methane mitigation, but these are a narrow spectrum and species-specific activity (Pulendran and Ahmed, 2006). The present approach is used to predict new anti-methanogenic targets from its precisely annotated operome that resolves the current demand for veterinary therapeutics.

## Materials and Methods

### Dataset Preparation

**FIGURE 1:**
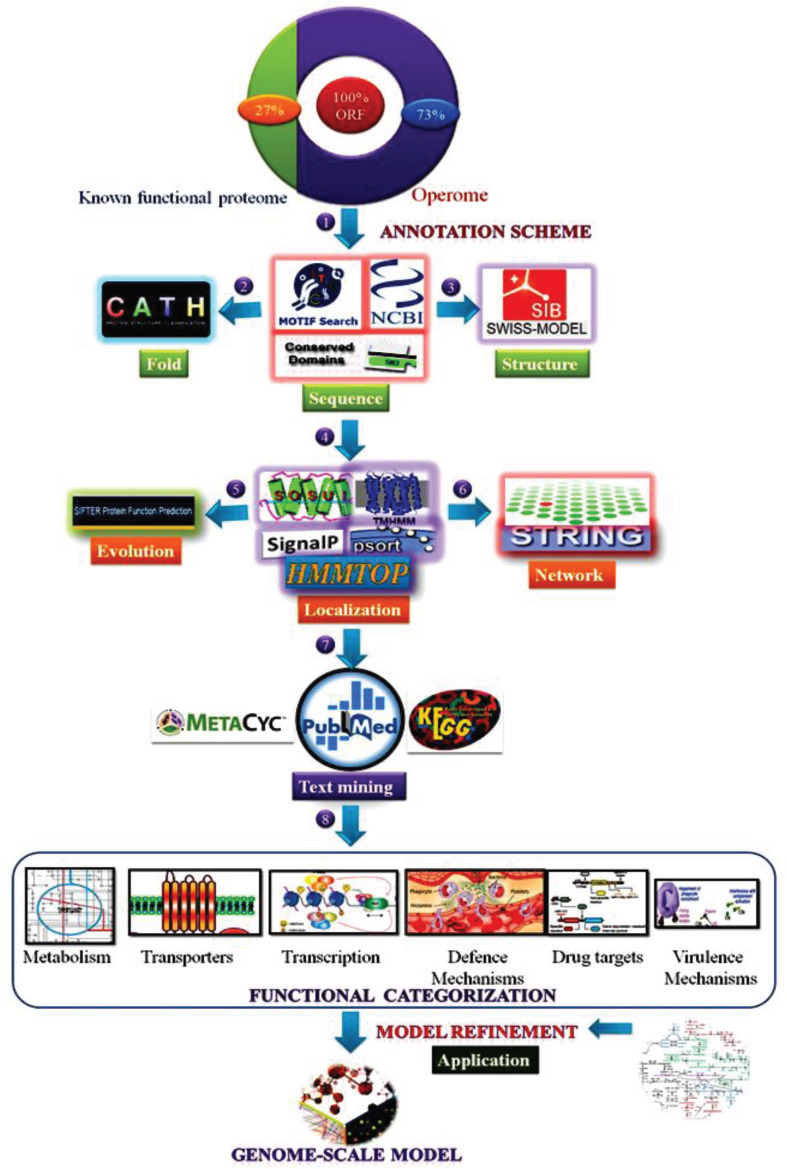
Experimental workflow of a combined bioinformatics approach employed for functional annotation of operome from MRU.

We retrieved protein sequences of 756 HPs in the MRU genome from the National Centre for Biotechnology Information (NCBI)^1^ and Kyoto Encyclopedia of Genes and Genomes (KEGG) (Kanehisa et al., 2018) using a simple text mining approach (Le and Huynh, 2019; Le et al., 2019). We used broad ranges of source types such as keywords, “*hypothetical proteins*, *unknown*, *uncharacterized*, and *putative*” to retrieve the protein sequences from the NCBI and KEGG (Chellapandi et al., 2017b). The FASTA sequences of all HPs were taken separately to carry out sequence analysis. For functional annotation and assignment of MRU operome, we used six different prediction tasks as detailed below (Figure 1). The overall information about similar or identical functions of HPs predicted from each task was manually evaluated to reasoning out the functional assignment of operome. The prediction tools used for each functional annotation were more robust and confident for our analysis similar to the previous works on archaeal and bacterial operome (Prathiviraj and Chellapandi, 2019; Sangavai et al., 2020). E-value is the number of expected hits of a similar score that could be found just by chance. Like *p*-value, we used e-value for the scoring of each prediction from the dataset and represented in Supplementary Data.

### Conserved Motif Analysis

A motif is a short segment of a protein sequence or structure, which may be conserved in a large number of different proteins. It can be used to determine the function or conformation of a protein. The conserved motifs in each protein were searched out against the KEGG-Motif search tool^2^, InterProScan (Quevillon et al., 2005), and Pfam library (Finn et al., 2016). To improve the lineament of prediction, cut off value was set as 10^–5^ and DUF (domains with unknown functions) were removed from the dataset. We found motif similarity hits for 756 HPs out of which 257 HPs were chosen for further analysis.

### Conserved Domain Analysis

Conserved domains in each protein were identified by the NCBI-CDD v3.16 search tool using the position-dependent weight matrices. Additionally, composition-based statistics adjustment was used to remove low complexity composition for statistical significance using the RPS-BLAST version 2.2.28 (Marchler-Bauer et al., 2015). The query sequence was compared with domain architecture and profiles in the domain databases, after that, the compositionally biased conserved region was identified by the SMART (Letunic et al., 2012). The PROSITE profile was scanned for detection of the protein domains, families, and functional sites and associated patterns in the protein sequence using ScanProsite (de Castro et al., 2006). The probable function of HPs was predicted with the InterPro database based on the domain and important sites in the sequences (Finn et al., 2016).

### Structural Analysis

The secondary structural elements (helix, sheets, extended coil, and loops) in each protein were predicted from the sequences using SOPMA (Geourjon and Deléage, 1995). We identified structural and functional characteristics by PSI-BLAST similarity searching against the protein data bank^3^ (Altschul et al., 1997). The sequence similarity hits were selected for finding the alignment of functional residues of a protein of known function with the sequence of HPs using ClustalW (Thompson et al., 2002). Fold assignment, target-template alignment, model building, and model evaluation were carried out with the Swiss Model (Biasini et al., 2014). QMEAN was a composite scoring function describing the major geometrical aspects of protein structures as described below.

$$S_{{\text{weighted}}_{\text{average}}\,\left(x\right)}=\frac{\Sigma_{i}\,\left({\text{GDT}}_{\mathrm{TS}\,\left(x,i\right)}^{*}\,\mathrm{QMEAN}\,\left(i\right)\right)}{\Sigma_{i}\,\mathrm{QMEAN}\,\left(i\right)}$$

where, the GDT_TS score as the target function. We evaluated the structural quality and accuracy of the resulted homology models based on the potential function as below (Benkert et al., 2008).

$$\mathrm{QMEAN5}\text{score}=0.3\times {\text{Score}}_{\mathrm{torsion}\,3-\mathrm{residue}}+0.17\times {\text{Score}}_{\mathrm{pairwise}\,C\,\beta /\mathrm{SSE}}+0.7\times {\text{Score}}_{\mathrm{solvation}\,C\,\beta }+80\times {\text{Score}}_{\mathrm{SSE}\,\mathrm{PSPIRED}}+45\times {\text{Score}}_{\text{ACCpro}}$$

### Evolutionary Trace Analysis

The evolutionary relationships to deduce the functionality of operome were inferred using the SIFTER (Radivojac et al., 2013). It was used to predict the protein function and Gene ontology term using the following confidence score.

$$\mathrm{Sg}\,\left(f\right)=1-\prod_{i=1}^{k}\left(1-\mathrm{Sg}\,\left(f\right)\right)$$

where, Sg(f) confidence score as the default prediction for a query protein g, Sg_*i*_(f) is the probability domain has function f (Sahraeian et al., 2015).

### Analysis of Physicochemical Properties

The physiochemical properties including molecular weight, theoretical pI, instability index, aliphatic index, and grand average of hydropathicity of HPs were predicted from their sequences using the Expasy’s Protparam server^4^. The instability index provides an estimate of the stability of a protein. An instability index <40 is predicted to be stable, and a value >40 is predicted to be unstable. The instability index uses the following weight values.

$$\text{II}=\left(\frac{10}{L}\right)*\sum_{\text{i}=1}^{\text{i}=\text{L}-1}\text{DIWV}\left(\text{x[i}\right]\text{x}\left[\text{i}+1\right])$$

where, L is the length of the sequence, DIWV(x[i]x[i+1]) is the instability weight value for the dipeptide starting in position I (Guruprasad et al., 1990). The aliphatic index of a protein is defined as the relative volume occupied by aliphatic side chain amino acids using the following equation.

Aliphatic⁢index⁢X⁢(Ala)+a*X⁢(Val)+b*(X⁢(Ile)+X⁢(Leu))

Where, X(Ala), X(Val), X(Ile), and X(Leu) are mole percent (100 X mole fraction) (Ikai, 1980). The GRAVY value for a protein is calculated as the sum of the hydropathy values of all of the amino acids divided by the number of residues in the sequence (Kyte and Doolittle, 1982).

### Analysis of Protein Subcellular Localization

The subcellular localization of every protein was predicted with PSORTb version 3.0.2 based on the hydrophobicity index of amino acids (Yu et al., 2010). The propensity of a protein for being a membrane protein was predicted by SOSUI 2.0 based on the physicochemical parameters (Mitaku et al., 2002). The transmembrane helix and topology of each protein were detected by the TMHMM 2.0 (Krogh et al., 2001) and HMMTOP (Tusnády and Simon, 2001) using the Hidden Markov Model. The signal peptide and location of the cleavage site in the peptide chain were predicted with the SingnalP 4.0 based on a neural network model (Petersen et al., 2011).

### Literature Search

The literature survey is the stepping-stone and an essential skill toward the accomplishment of structural and functional analysis provides of proteins (Hubbard and Dunbar, 2017). A process of uncovering useful knowledge from a collection of data from bioinformatics and literature databases is referred to as a knowledge-based discovery (Chellapandi et al., 2017b). Functional assessment of operome was strengthened by extracting relevant experimental supports from available literature in NCBI-PubMed^5^. A maximum confidence score was set as 12 levels (6 levels from predictions and 6 levels from the literature mining) in which 50% score systematically enumerated and assigned from overall prediction approaches. The rest of them was assigned by manual annotation based on the strength of the literature validation. For example, if the predicted function is similar or identical in all prediction approaches, a maximum confidence score will be assigned as 6. The literature-based confidence score for each predicted function of HPs assigned as; 6- MRU, 5- Phylogenetic neighbors, 4- Methanogens, 3- Archaea, 2- Bacteria, and 1- Eukaryotes. We have set a confidence score interval as 3–6 for both computational prediction and biological knowledge base and then neglected the predicted function of a protein with a low confidence score (<3).

### Functional Categorization

We classified the predicted function of HPs based on conserved domain, protein fold, family, and biological function using the CATH database (Knudsen and Wiuf, 2010). The genome-wide analysis was performed to identify the order of gene clusters covering the predicted function of HPs using a genomic context approach (Yellaboina et al., 2007). Gene-neighborhood or adjutant genes were identified by exploring the MRU genome in the KEGG database. Metabolic information of HPs was collected from the MetaCyc (Metabolic Pathways from all Domains of Life) database (Caspi et al., 2014). The resulted data were used to assign the functions of hypothetical proteins of the understudied genome. The overall structural and functional information was manually analyzed to categorize the molecular involvement of HPs in respective metabolic subsystems and the cellular process of the understudied organism.

## Results

### Functional Classification and Categorization

**FIGURE 2:**
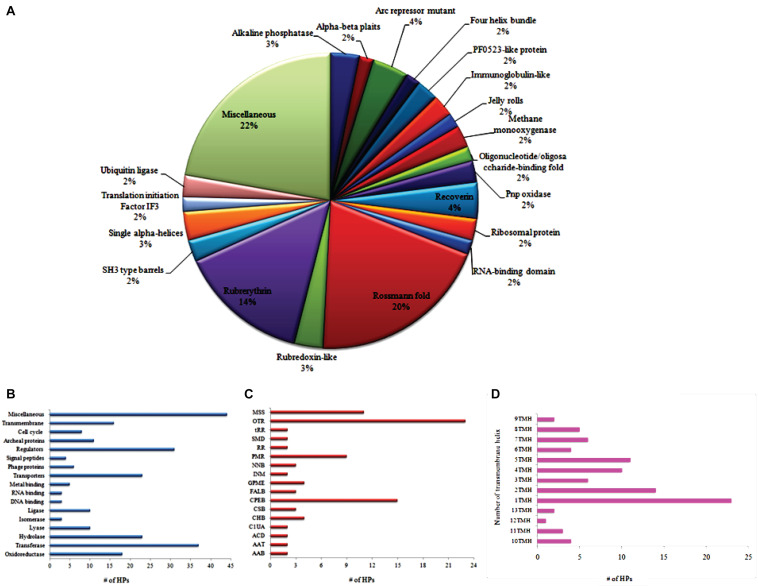
Functional classification of MRU operome based on the protein fold **(A)**, functional category **(B)**, subpathway systems **(C)**, and transmembrane topologies **(D)**. AAB, Amino acid biosynthesis; AAT, Aminoacyl-tRNA charging metabolic clusters; ACD, Aromatic compounds degradation; C1UA, C1 Compounds utilization and assimilation; CHB, Carbohydrates biosynthesis; CSB, Cell structures biosynthesis; CPEB, Cofactors, prosthetic groups, electron carriers biosynthesis; FALB, Fatty acid and lipid biosynthesis; GPME, Generation of precursor metabolites and energy; INM, Inorganic nutrients metabolism; NNB, Nucleosides and nucleotides biosynthesis; PMR, Protein-modification reactions; RR, RNA-reactions; SMD, Secondary metabolites degradation; tRR, tRNA reactions; OTR, Other reactions.

All predicted protein functions were classified and categorized according to their protein folds, molecular function, subsystems, and transmembrane topologies as shown in Figure 2. About 20% of operome encompasses a Rossmann fold consisting of a nucleotide cofactor binding domain of some NAD^+^-dependent dehydrogenases, in particular to ribonucleases (Barbas et al., 2013). Fourteen percent of operome belongs to rubrerythrin that constitutes non-haem iron proteins. This functional fold is responsible for oxidative stress protection in anaerobic bacteria and archaea (Prakash et al., 2018). The arcR repressor mutant fold occupies 4–5% of operome, which performs the functions of small homodimeric proteins involved in transcriptional regulation by sequence-specific DNA binding (Vershon et al., 1986; Homa and Brown, 1997). MRU operome contains phoA fold (3–4%) that fused with the cell surface glycoprotein signal sequence similar to *Haloferax volcanii* (Kandiba et al., 2013). It indicates the importance of some protein folds for conferring oxidative tolerance and cell wall assembly. We found 91 HPs involving in the metabolic reactions with a confidence score >5. A total of 23 HPs is entailed in the small molecule reactions and 15 HPs required for the biosynthesis of cofactors, prosthetic groups, and electron carriers. About 9 HPs are essential to the protein modification reactions whereas 4 HPs contributed to the formation of precursor metabolites for the energy-driven process of this organism. Approximately 50% of drug targets are transmembrane proteins as they play many roles in transport, cell signaling, and energy transduction processes (Terstappen and Reggiani, 2001). We predicted 91 HPs having transmembrane helixes based on their conservation of membranous helix ratios. The α-helix bundle and the β-barrel are predicted as fold classes in many membrane proteins. Archaeal transmembrane proteins have two or more α-helixes consisting of hydrophobic amino acids.

### Operon-Like Organization

**FIGURE 3:**
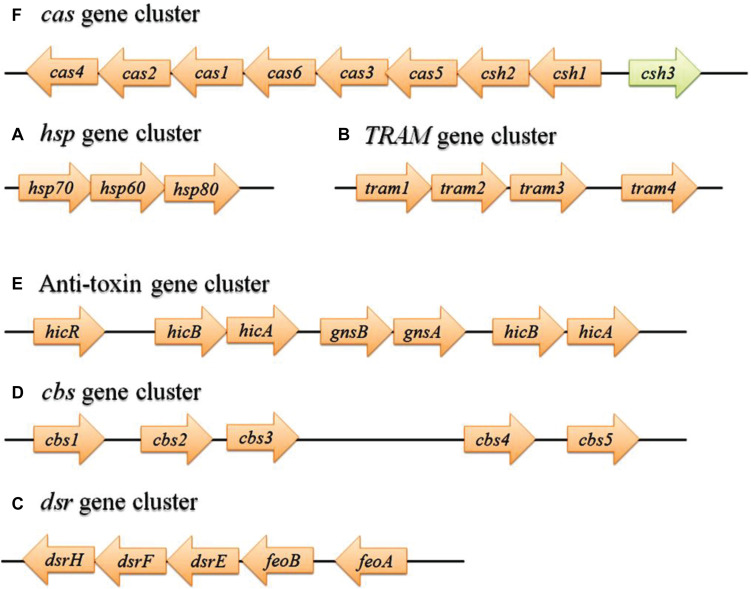
Detection of gene clusters from MRU operome responsible for protein folding **(A)**, cold adaptation **(B)**, sulfite tolerance **(C)**, binding with adenosyl groups **(D)**, degradation of the labile antitoxin **(E)**, and defense/virulence system **(F)**. The green arrow represents a gene with a known function. hsp, Heat shock protein; TRAM, RNA modification protein; dsr, Dissimilatory sulfate reductase; cbs, cystathionine beta-synthase; cas, CRISPR-associated gene.

The genome-wide analysis discovered 32 coding genes for HPs, which are all clustered separately, form 6 operon-like organizations (*hsp, TRAM, dsr, cbs, anti-toxin*, and *cas*) in the MRU genome (Figure 3). Molecular chaperones such as hsp70, hsp60, and hsp80 resemble some bacterial genomes than the eukaryotic homologs (Gaywee et al., 2002). The *hsp* gene cluster is essential for chaperone-assisted protein folding in Achaea (Dokland, 1999; Benaroudj and Goldberg, 2000; Large et al., 2009). The assimilatory sulfite reductase (*dsrHFEBA*) gene cluster detected from this genome provides the importance for the oxidation of accumulated intracellular sulfide and thiosulfate in the diverse environmental niche. The presence of *cbs, anti-toxin*, and *cas* gene clusters confers host defense response (innate immunity) to this organism against foreign genetic elements in the rumen ecosystem (Louwen et al., 2014; Chellapandi and Ranjani, 2015). The anti-toxin system plays a vital role in toxicity neutralization (Unterholzner et al., 2013).

### Cell Division Systems

In this study, we assigned the function of 9 HPs contributing a major role in the cell cycle process in which 8 HPs have shown new functions to this organism (Table 1). AAA^+^ ATPase, cell division inhibitor, cell division control protein, DNA replication protein 6-2, and structural maintenance of chromosomes protein-1 is highly conserved within the archaeal domain and performs archaeal-specific cell cycle process, DNA repair, and replication fidelity (Kalliomaa-Sanford et al., 2012; Grogan, 2015). A proteasome is a central player in energy-dependent proteolysis and forms a nano-compartment where proteins are degraded into oligopeptides by processive hydrolysis. The 20S proteasome is a catalytic core responsible for this processing. AAA^+^ ATPase plays several roles in mediating energy-dependent proteolysis by the proteasome (Forouhar et al., 2011; Maupin-Furlow, 2013). Moreover, it contains a P-loop motif involved in the origin of recognition during DNA replication initiation even if conventional C-terminal winged-helix DNA-binding elements lacked (He et al., 2008).

**TABLE 1:** Functional annotation of operome involved in the cell division process of MRU.

Locus tag	Assigned function	Gene
0080| 0744| 0939| 1172| 1932	AAA^+^ ATPase	*atad3A*
0647	Cell division inhibitor	*sepF*
1346	Cell division control protein	*minE*
1419	DNA replication protein 6-2	*cdc6-2*
1654	Structural maintenance of chromosomes protein 1	*smc1*

### Transcriptional Regulatory Systems

A total of 26 HPs predicted as functional candidates in which 20 HPs have shown new functions to the transcriptional regulation process of this organism (Table 2). Transcriptional regulatory proteins identified from MRU operome can express a set of proteins that protect cellular proteins against a sudden heat-shock stress, copper and arsenic toxicities, protein folding, and nitrogen starvation (Thieringer et al., 1998; Giaquinto et al., 2007; Chang et al., 2014; Prathiviraj and Chellapandi, 2020a, b). Bro N-terminal domain protein has an N-terminal domain with ALI motif that influences host DNA replication and/or transcription (Makarova et al., 2009). HrcA repressor contains a motif of winged helix-turn-helix transcription repressor. It controls the transcription of heat-shock repressor proteins and protects cellular proteins from being denatured by heat (Liu et al., 2005; Prathiviraj and Chellapandi, 2020b). Hsp70 and Hsp80 from MRU operome perform renaturation of luciferase similar to that found in *M. mazei* (Zmijewski et al., 2004). Hsp60s are more similar to the type II chaperonins found in the eukaryotic cytosol involved in macromolecular assembly and protein folding (Large et al., 2009). TRAM protein regulates the RNA chaperone activity that is essential for MRU to grow and survive in a cold environment (Zhang et al., 2017).

**TABLE 2:** Functional annotation of operome involved in thetranscriptional regulatory process of MRU.

Locus tag	Assigned function	Gene
0757	Bro N-terminal domain protein	*dxs*
0349	Nitrogen repressor	*nrpR*
1052	Heat-inducible transcriptional repressor	*hrcA*
1099	Translation initiation factor 3	*tif3*
1366| 2156	Arsenical resistance operon repressor	*arsR*
1862	Copper-sensing transcriptional repressor	*csoR*
0488| 0490| 0499| 0658| 0764| 0780| 0790| 0801| 0930| 1131| 1147| 1150| 1364| 1590| 1796	Transcription factor	*tf2B*
1185	Cold shock protein	
1108	DEAD/DEAH box helicase	*polB*
0877	Preprotein translocase	*secY*

### Biosynthesis of Macromolecules

We predicted the function of 20 HPs exhibiting new metabolic roles in this organism and the rest of 76 HPs has shown known functions (Table 3 and Supplementary Table S1). Saccharopine dehydrogenase (NAD/P, L-lysine-forming) (*lysA*) and succinylglutamate desuccinylase (*astE*) genes identified from MRU operome, which are responsible to mediate the biosynthesis of L-lysine and L-glutamate. LysA protein contains a motif of LOR/SDH bifunctional conserved region that converts L-saccharopine into L-lysine via l-α-aminoadipate pathway (Xu et al., 2007). Cheng et al. (2010), revealed a cross-talk between fungi and methanogens which may occur in host animals since the l-α-aminoadipate pathway is very specific to fungi. The second enzyme transforms N_2_-succinylglutamate into succinate and glutamate. Therefore, both enzymes proposed to be involved in amino acid biosynthesis of MRU as reported earlier on other methanogens (Enzmann et al., 2018).

**TABLE 3:** Functional annotation of operome involved in differentmetabolic subsystems of MRU.

Locus tag	Assigned function	EC	Gene
**Biosynthesis**			
*Amino acids biosynthesis*			
1696	Carbamoyl-phosphate synthase (glutamine-hydrolyzing)	6.3.5.5	*carB*
1737	Saccharopine dehydrogenase (NAD/P, L-lysine-forming)	1.5.1.7| 1.5.1.8	*lys1*
**Aminoacyl-tRNA charging metabolic clusters**			
0488| 0490| 0499| 0764| 0780| 0790| 0801| 1131| 1147| 1150| 1364| 1590| 1796	Methionine—tRNA ligase	6.1.1.10	*metG*
1493	Tryptophanyl-tRNA synthetase (Membrane bound)	6.1.1.2	*trpS*
**Carbohydrates and Cell structures biosynthesis**			
1418	dTDP-4-amino-4,6-dideoxygalactose transaminase	2.6.1.59	*rffA*
1886	Glycogen Phosphorylase	2.4.1.1	*glgP*
1469	UDP-glucose 4-epimerase	5.1.3.2	*galE*
1462	Pantothenate synthase	6.3.2.1	*panC*
0480	Pyruvate kinase	2.7.1.40	*pykA*
**Cell structures biosynthesis**			
1065	CDP-glycerol glycerophosphotransferase	2.7.8.12	*tagF*
1589| 1957	Thiamine monophosphate synthase	2.7.7.39	*tagD*
**Cofactors, Prosthetic groups, Electron carriers biosynthesis**			
2219	Cobalamin biosynthesis protein CbiB	6.3.1.10	*cbiB*
0947	Coenzyme F420-0:L-glutamate ligase	6.3.2.31	*cofE*
1116	CTP: Molybdenum cofactor cytidylyltransferase	2.7.7.76	*–*
1450	Energy-converting hydrogenase B subunit O	1.6.5.3	*ehbO*
0776| 0785	Gamma-glutamyl cyclotransferase	2.3.2.4	*ykqA*
1937	Glutathione peroxidase	1.11.1.9	*gpxA*
0035	NUDIX hydrolase	3.6.1.22	*nadM*
0596	4-Hydroxybenzoate octaprenyltransferase	2.5.1.39	*ubiA*
1550	5-Formyltetrahydrofolate cyclo-ligase activity	6.3.3.2	*mthfs*
1831	Dihydroneopterin aldolase	4.1.2.25	*folB*
1209	Nicotinate-nucleotide pyrophosphorylase [carboxylating]	2.4.2.19	*nadC*
0277	NUDIX hydrolase	3.6.1.22	*nudC*
2172	Riboflavin kinase	2.7.1.161	*ribK*
0432	Tocopherol cyclase	5.5.1.24	*vte1*
1728	Phosphomevalonate decarboxylase	4.1.1.99	*pmd*
**Fatty acid and lipid biosynthesis**			
0460	Dolichol kinase	2.7.1.108	*dolk*
1693	Integral Membrane bound Phosphatidate cytidylyltransferase	2.7.7.41	*cdsA*
**Metabolic regulators biosynthesis**			
0939	6-Phosphofructo-2-kinase| Fructose-2,6-bisphosphate 2-phosphatase	2.7.1.105| 3.1.3.46	*pfkfb3*
0393 (K18532 adenylate kinase [EC:2.7.4.3])	Adenylate kinase	2.7.4.3	*adk*
**Nucleosides and nucleotides biosynthesis**			
0425	L-Threonylcarbamoyladenylate synthase	2.7.7.87	*yrdC/sua5/ywlC*
1890	Phosphoribosylaminoimidazole carboxylase	4.1.1.21	*purE*
0720	Uridylate kinase (DNA binding protein)	2.7.4.22	*pyrH*
**Catabolism**			
*Alcohols degradation*			
0528	Coenzyme B12-dependent diol dehydrase	4.2.1.28	*pduC*
**Amino acids degradation**			
2016| 0381	Succinylglutamate desuccinylase	3.5.1.96	*astE*
**Aromatic compounds degradation**			
1622	4-Carboxymuconolactone decarboxylase	4.1.1.44	*pcaC*
0476	Phenylacetate-CoA oxygenase	1.14.13.149	*paaJ*
0313	Pyrogallol hydroxytransferase	1.97.1.2	*athL*
**C_1_ Compounds utilization and assimilation**			
2132	Bifunctional formaldehyde-activating enzyme	4.2.1.147/4.1.2.43	*fae-hps*
1013	Phosphogluconate dehydrogenase (NAD+-dependent, decarboxylating)	1.1.1.343	*gntZ*
**Inorganic nutrients metabolism**			
1280| 1936	NADPH-dependent FMN reductase	1.5.1.38	*ssuE*
0224| 0376	Phosphonoacetate hydrolase (membrane bound)	3.11.1.2	*phnA*
**Secondary metabolites degradation**			
1330	Carbohydrate kinase (Integral membrane-bound)	2.7.1.4	*pfkB*
2120	Quercetin dioxygenase	1.13.11.24	*qodI*
**Macromolecule modification**			
0421	Alpha-2,3-sialyltransferase	2.4.99.4	*siat4a*
**Small molecule reactions**			
1938	Arsenate Reductase (Thioredoxin)	1.20.4.1	*arsC*
0134	Type I restriction-modification system M subunit HsdM	2.1.1.72	*hsdM*
0674| 1683| 1749	Succinate dehydrogenase (quinone)	1.3.5.1	*sdh*
1013	Phosphogluconate dehydrogenase (NAD+-dependent, decarboxylating)	1.1.1.343	*gntZ*
2194	2-Enoyl-CoA Hydratase	3.4.21.92	*clpP*
0747	2-Polyprenylphenol 6- hydroxylase	1.14.13.-	*ubiB2*
0202	Aconitate hydratase	4.2.1.3	*acnA*
2180| 2184| 2185	Acyltransferase	2.3.1.13	*glyat*
0496	ATP pyrophosphatase	3.6.1.8	*thiI*
0062| 0063| 1113| 1172	ATP-dependent DNA helicase	3.6.4.12	*ashA*
2196	Choloylglycine hydrolase	3.5.1.24	–
0156| 0041	DNA binding E3 SUMO-protein ligase	6.3.2.-	*piaS4*
0174 (K09723 DNA replication factor GINS)	DNA primase small subunit	2.7.7.-	*priA*
2069	DNA-3-methyladenine glycosylase	3.2.2.20	*tag*
1108| 2173	DNA-directed DNA polymerase	2.7.7.7	*polB*
1660| 1699| 1734	Flavin reductase	1.5.1.36	*hpaC*
1442	Geranylgeranyl reductase	1.3.1.83	*chlP*
1290| 1291	Lincosamide nucleotidyltransferase	2.7.7.-	*inuA*
0930	Manganese-dependent inorganic pyrophosphatase	3.6.1.1	*ppaC*
0223	Membrane-bound O-acyltransferase	2.3.1.-	*rimL*
1242	Nucleoside Triphosphate Pyrophosphohydrolase	3.6.1.8	*mazG*
1605| 0049	Nucleotide diphosphatase	3.6.1.9	*ENPP*
2146	Oligosaccharyl transferase	2.4.99.18	*STT3*
1588	Succinylglutamate desuccinylase/aspartoacylase	3.5.1.15	*aspA*
0100	Peptidoglycan-associated polymer biosynthesis	2.-.-.-	*csaB*
1555	Pseudouridine-5’-monophosphatase	3.1.3.-	*HDHD1*
1964	Sterol 3-beta-glucosyltransferase (Phosphorylating)	2.4.1.173	*–*
1631	UDP-N-acetylglucosamine 2-epimerase (non-hydrolyzing)	5.1.3.14	*wecB*
0835	*von* Willebrand/Integrin A Domains	3.6.4.-	*hepA*
**Protein-modification reactions**			
1344	Lysine carboxypeptidase	3.4.17.3	*CPN1*
1375	Membrane-bound dolichyl-phosphate-mannose-protein mannosyltransferase	2.4.1.109	*pomT*
0791	Methylated-DNA—[protein]-cysteine S-methyltransferase	2.1.1.63	–
1884	Nucleotide-activated 6-deoxyhexose biosynthesis	2.4.1.109	*pomT*
2158	Putative pyruvate formate-lyase	1.97.1.4	*pflX*
1801| 1867	Ribosomal-protein-alanine N-acetyltransferase	2.3.1.128	*rimI*
1389| 1514	S-Adenosyl-L-methionine-dependent methyltransferase	1.16.1.8	*mtrR*
1096	Serine/threonine protein kinase with TPR repeats	2.7.11.1	*bub1*
1563	Proteasome endopeptidase complex	3.4.25.1	*psmA*
1311| 0426	tRNA-splicing ligase	6.5.1.3	*rtcB*
**Energy metabolism**			
*Generation of precursor metabolites and energy*			
2214	Fuculose 1-phosphate aldolase	4.1.2.17	*fucA*
1894	Fumarate hydratase	4.2.1.2	*fumA*
			

The 2-enoyl-CoA hydratase catalyzes the second step in the physiologically important β-oxidation pathway of fatty acid metabolism in MRU (Agnihotri and Liu, 2003). Glycogen phosphorylase catalyzes the phosphorolysis of α-1, 4 glycosidic bonds in glycogen to yield glucose-1-phosphate for glycolysis (Rath et al., 2000). Interestingly, MRU operome has the ability to synthesis enterobacterial-like common antigen as it contains dTDP-4-amino-4, 6-dideoxygalactose transaminase (*rffA*). This enzyme catalyzes the conversion of TDP-4-keto-6-deoxy-D-glucose to TDP-D-fucosamine similar to the enterobacteria family (Meier-Dieter et al., 1990; Hwang et al., 2004). The presence of phosphatidate cytidylyltransferase (*cdsA*) provides evidence of the biosynthesis of archaeal-specific phospholipids. It catalyzes *sn*-glycerol 3-phosphate into an L-1-phosphatidylglycerol-phosphate precursor-like *Escherichia coli* (Carter et al., 1968). We found an AMMECR1 motif in phosphomevalonate decarboxylase from MRU operome, which converts (R)-mevalonate 5-phosphate to isopentenyl diphosphate in the mevalonate pathway, as reported in *Methanocaldococcus jannaschii* (Grochowski et al., 2006). Results of our study revealed that the MRU genome has shown a metabolic potential for the biosynthesis of enterobacterial-like common antigen, archaeal-specific phospholipids, and isopentenyl diphosphate, a precursor required for cell wall biogenesis.

### Cofactors, Prosthetic Groups, Electron Carrier Biosynthesis

We predicted the function of some HPs involving in the biosynthesis of coenzyme F_420_, flavin, and electron carriers in MRU. F_420_-0: L-glutamate ligase is a key enzyme identified from MRU operome, which converts multiple γ-linked L-glutamates to the polyglutamated F_420_derivative in the biosynthesis of coenzyme F_420_ (Li et al., 2003). As reported in bacteria and plants, MRU operome has diamino hydroxy phosphoribosyl aminopyrimidine reductase (*ribD*) that converts 2, 5-diamino-6-(5-phospho-D-ribosylamino)pyrimidine-4(3H)-one into 5-amino-6-(5-phospho-D-ribosylamino)uracil in flavin biosynthesis pathway (Garfoot et al., 2014). Cytidylyltransferase belongs to the NTP transferase superfamily encoded by *mocA* gene (mru_1116) of the MRU genome. It catalyzes the cytidylation of the molybdenum cofactor demanded many functional enzymes (Fay et al., 2015). Energy-converting hydrogenase B subunit O consists of a conserved motif of IHPPAH, which generates low potential electrons required for autotrophic CO_2_ assimilation as reported in *Methanococcus maripaludis* (Major et al., 2010).

### Aromatic Compounds Degradation Systems

Pyrogallol hydroxytransferase (*athL*) detected from MRU operome has a carboxypeptidase regulatory-like domain. It is involved only in the regulation of peptidase catalyzing the conversion of pyrogallol into phloroglucinol. Phloroglucinolcan stimulates the gut microbiota and decreases the partial pressure of H_2_ in the rumen. It suggests the capture of excess H_2_ generated from methanogenesis inhibition can be promoted by phloroglucinol utilization in the rumen (Martinez-Fernandez et al., 2017). Interestingly, we assigned a precise function to HP Mru_0476 as phenylacetate-CoA oxygenase in phenylacetate catabolic pathway. This enzyme converts phenylacetyl-CoA to a 2-(1, 2-epoxy-1, 2-dihydrophenyl) acetyl-CoA. Archaea harboring key genes of this pathway are some members of the Halobacteria, which may have acquired a multitude of bacterial genes (Kennedy et al., 2001; Notomista et al., 2003). As shown by our analysis, MRU can degrade pyrogallol and phenylacetate produced by gut microbial in ruminants (Martinez-Fernandez et al., 2017).

### Detoxification Systems

MRU operome plays a key role in formaldehyde, inorganic arsenate, and copper detoxification process. It contains 6-phosphogluconate dehydrogenase (*gntZ*) gene as homologous to methanotrophic bacteria such as *Methylophilus methylotrophus* and *Methylobacillus flagellates* (Chistoserdova et al., 2000). The presence of arsenate reductase (*arsC*) and Cu^+^-exporting ATPase (*copA*) provides a defense system to its cells against inorganic arsenate and copper toxicities (Liu et al., 2007).

### Macromolecule Modification Systems

MRU operome contains α-2, 3-sialyltransferase gene coding protein having a Rossmann fold with the architecture of the α-β complex. This enzyme catalyzes the transfer of sialic acid from CMP-N-acetyl-β-neuraminate to membrane proteins and lipids of the cell wall of MRU (Koga et al., 1993). Dolichyl-phosphate-mannose-protein mannosyltransferase is identified as carbohydrate carriers to transfer mannosyl residues to the hydroxy group of serine or threonine residues during the post-translational protein modification process of MRU (Podar et al., 2013).

### Membrane Transport Systems

We observed 16 HPs contributing to the transport systems of this organism (Supplementary Table S2). MRU operome encompasses genes coding for transporter proteins responsible for maintenance of metal homeostasis in particular to magnesium and manganese ions and uptake/export of vitamin, sulfite, and tricarboxylate (Winnen et al., 2003; Weinitschke et al., 2007; Hattori et al., 2007, 2009; Rodionov et al., 2009; Rosch et al., 2009; Mayer et al., 2012; Karpowich et al., 2015). The presence of PurR-regulated permease regulon and Na^+^/H^+^ antiporter protein carries out the exchange Na^+^ for H^+^ across the cytoplasmic membrane of archaea (Rimon et al., 2012). Cell-cell communication and intra-species electron transfer can be mediated by preprotein translocase predicted from its operome, as described for hydrogenotrophic methanogens and *E. coli* (Cooper et al., 2017). Translocation sheath protein has an N-terminal domain that mediates the translocation of SPI-2 TTSS effector proteins in MRU (Nikolaus et al., 2001).

### D-Gluconate Catabolic System

**FIGURE 4:**
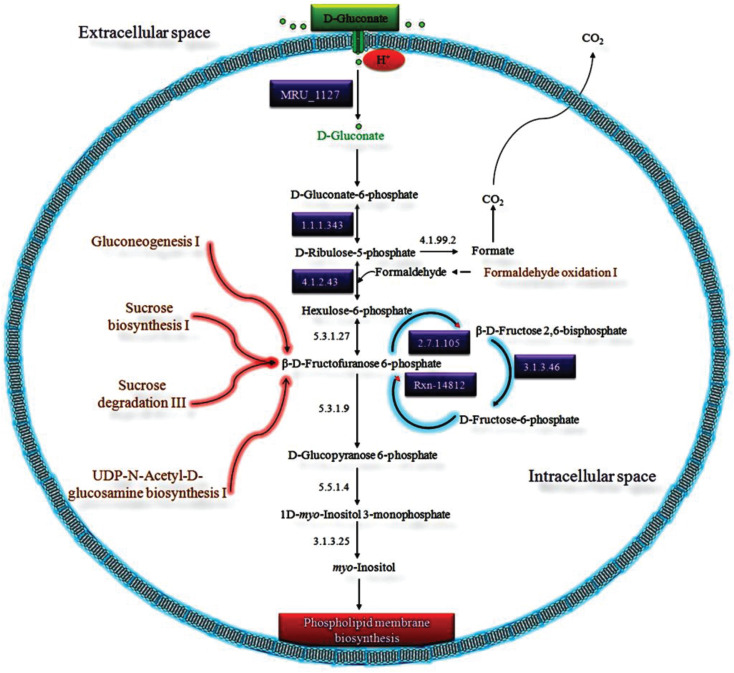
The proposed D-gluconate catabolic pathway in MRU was discovered from the functional annotation of its operome. D-Gluconate is imported into the cytoplasm by the predicted gluconate transporter (*gntP*) gene. It can be phosphorylated to D-gluconate-6-phosphate by D-gluconate kinase (*gntK*), which is then converted to D-ribulose-5-phosphate by the catalytic action of NAD^+^-dependent phosphogluconate dehydrogenase (*gntZ*). D-Ribulose-5-phosphate is next oxidized to hexulose-6-phosphate by 3-hexulose phosphate synthase (*hxlA*) and converted into β-D-fructofuranose 6-phosphate with phospho-3-hexuloisomerase (*phi1*). The 6-phosphofructose 2-kinase phosphorylates β-D-fructofuranose 6-phosphate into β-D-fructose 2, 6-bisphosphate, which then interconverted from D-fructose-6-phosphate to β-D-fructofuranose 6-phosphate by fructose-2, 6-bisphosphate 2-phosphatase. In an alternative way, β—D-fructofuranose 6-phosphate is phosphorylated to D-glucopyranose 6-phosphate by 6-phosphofructo-2-kinase. Glucopyranose 6-phosphate is converted to 1D-*myo*-inositol 3-monophosphate by D-glucose 6-phosphate cycloaldolase (*ino1*) and reduced to *myo*-inositol by inositol-phosphate phosphatase (*suhB*).

As shown by our analysis, we proposed a putative D-gluconate catabolic pathway exclusively present in MRU for the biosynthesis of archaeal membrane phospholipids (Figure 4). The presence of six HPs with predicted functions evidences the existence of this pathway in this organism. Klemm et al. (1996), identified a gntP gene to be involved in gluconate uptake by *E. coli*. *Haloferax volcanii* contains a DeoR/GlpR-type transcription factor, which has shown its potential role as a global regulator of sugar metabolism and to cotranscribe with the downstream phosphofructokinase (*pfkB*) gene (Rawls et al., 2010). As similar to *Pseudomonas aeruginosa*, MRU operome has D-gluconate kinase gene despite a membrane-bound D-gluconate dehydrogenase gene to synthesize phospholipids (Matsushita et al., 1979; Schlictman et al., 1995; Kulakova et al., 2001). As similar to archaea, the utilization of gluconate in MRU leads to a branch point for two central metabolic pathways: the Entner-Doudoroff pathway and phospholipids biosynthesis (Bräsen et al., 2014).

## Discussion

The function of operome is obscure and quite unsettling in prokaryotic genomes. Understanding important knowledge gaps in the unknown function of operome can unravel their cellular and molecular mechanisms. The functionality of proteins with unknown function have been identified, characterized, and validated with a broad spectrum of genetic and biochemical experiments (Mills et al., 2015). Several computational methods have been used to describe the physiological states of methanogens from the predicted functions of operome (Chellapandi and Prisilla, 2018; Prathiviraj and Chellapandi, 2019). There are several functional measures (structural and functional motifs) to be considered for computational predictions of operome from available microbial genomes. The present study employed to collect comprehensive information derived from sequence similarity, conserved domain, motif, structure, fold, protein-protein interaction, subcellular localization, phylogenetic inference, and gene expression profile as the predictive measures to assign a precise molecular function to MRU operome. Collective information of them provides a hint to predict some distinct motifs and annotate the function of each protein accurately for studying growth physiology in the rumen ecosystem.

Generally, the protein sequence is less conserved than the tertiary structure of a protein (Illergård et al., 2009). In this study, experimentally solved structures and accurate protein folding offered the major importance to deduce some level of a functional description of a protein, as described by Nealon et al. (2017). Characterization of binding motifs and catalytic cores present in the proteins and functional categorization in the cell has been achieved by using the predictive measures derived from overall proteome information (Shapiro and Harris, 2000). Many protein domains have unknown functions, but they may contribute to the metabolic regulation of organisms (Kotze et al., 2013). It implied the possibility of finding a new domain and motif as well as discovers additional protein pathways and cascades from functionally annotated operome (Ijaq et al., 2015). Functional prediction and assignment of prokaryotic operome have been either only sequence-based or structure-based strategies. In our study, a combination of bioinformatics tools with 6 different prediction schemas and additional literature evidence with a 6-level confidence score was applied to improve the prediction accuracy of our functional assignment (Figure 1). Compared to earlier functional prediction approaches, our approach provides a strong emphasis to reveal its metabolic subsystems and cellular mechanisms from the assigned function of operome.

The mechanisms of molecular pathogenesis and virulence of many pathogenic organisms and drug targets discovery are being considered an accurate prediction of operome function as an important biological knowledgebase (Amavisit et al., 2003; Lamarche et al., 2008; Kumar et al., 2014). Several bioinformatics tools have been utilized for functional prediction of operome from different pathogenic organisms (Kumar et al., 2014, 2015; Singh et al., 2017; Shrivastava et al., 2017). It clearly described that all of them are pathogenic organisms but no reports on rumen methanogens yet. It was the first computational study to characterize the function of MRU operome, a potential methanogen for enteric methane emission in the ruminants via enteric fermentation.

The Rossmann was a novel and ancient fold found in 5, 10-methenyltetrahydromethanopterin hydrogenase, a key enzyme of hydrogenotrophic methanogenesis. It explains the possibility of hydrogenotrophic lifestyle in MRU, as described by Leahy et al. (2010). The reduction potentials of rubredoxin fold-containing proteins are known to be involved in biochemical processes including carbon fixation, detoxification, and fatty acid metabolism (Prakash et al., 2018; Prathiviraj and Chellapandi, 2020). Cofactors or other prosthetic groups are more attractive to stimulate enzyme activity in hydrolytic reactions of archaea. Transmembrane helixes are generally independently stable in a membrane or membrane-like environment, which are important for signal recognition, transport phenomena, energy translocation, and conservation in the living cell (von Heijne, 1988; Jennings, 1989). Concerning the functional importance, we classified and categorized the function of MRU operome in this study.

In this study, six operon-like clusters were identified from MRU operome. The functions of predicted gene clusters were contributed in chaperone-assisted protein folding, host defense response, and toxicity neutralization of MRU. Some transcriptional regulatory systems predicted from its operome have shown to protect cellular proteins against sudden heat-shock stress, nitrogen limitation, and heavy metal homeostasis. MRU genome contains many pathway holes, which hinder its accurate metabolic reconstruction at the genome-scale. In our study, we detected some key genes missing in the metabolic network of this organism. Consequently, complete metabolic subsystems were annotated for the biosynthesis of L-lysine, L-glutamate, enterobacterial-like common antigen, archaeal-specific phospholipids, and isopentenyl diphosphate. MRU operome can produce coenzyme F_420_ and flavin and electron carriers. Cell wall lipids and membrane proteins have been synthesized from the function of some HPs through macromolecule modification reactions. This organism has well-established transporter systems to maintain metal homeostasis and uptake/export of vitamin, gluconate, sulfite, and tricarboxylate. D-Gluconate catabolic pathway was uniquely discovered from MRU operome for the biosynthesis of archaeal membrane phospholipids.

## Conclusion

The functional assignment of operome is a mandatory process for a better understanding of the metabolic and molecular processes of this organism. The predicted functional properties of its operome afford us not only for new structural information but also for new molecular functions essential for the lifestyle in the rumen ecosystem. A major operome covers all functional counterparts needed to perform diverse metabolic pathways and regulatory processes. Some imperative physiological functions (oxidative stress, archaeal-specific membrane phospholipids, etc.) of this organism are revealed from this study. The genome-neighborhood analysis found six main gene clusters (hsp, tram, dsr, cbs, anti-toxin, and gas), which are contributed to the energetic metabolism and defense systems. MRU operome contains 119 metabolic enzymes with 18 sub-pathways and 25 binding proteins that recognize the DNA, RNA, metal, and membrane for cellular function. Interestingly, we discovered a putative D-gluconate catabolic pathway for the biosynthesis of archaeal-specific membrane phospholipids. Several virulence-associated and vaccine targeted proteins have been identified from MRU operome. It suggests the development of new methane mitigation interventions that target the key metabolic proteins to reduce methane emissions in ruminants. Functional prediction and assignment of its operome are thus very important to comprehend the cellular machinery at the systems-level for anti-methanogenic compounds discovery. Nevertheless, all of our predicted functions of its operome should be evaluated and validated experimentally with protein expression and purification, crystallization, and structure determination studies.

## Data Availability Statement

The original contributions presented in the study are included in the article/Supplementary Material, further inquiries can be directed to the corresponding author/s.

## Author Contributions

PC: research design, concept, and manuscript writing. MB: data preparation and analysis. NS: data analysis and manuscript revision. All authors contributed to the article and approved the submitted version.

## Conflict of Interest

The authors declare that the research was conducted in the absence of any commercial or financial relationships that could be construed as a potential conflict of interest.

## Footnotes

1 http://www.ncbi.nlm.nih.gov

2 http://www.genome.jp/tools/motif/

3 www.rcsb.org/

4 http://web.expasy.org/protparam/

5 https://www.ncbi.nlm.nih.gov/pubmed/

## Supplementary Material

Supplementary Table 1 Functional annotation of operome involved in diverse metabolic systems of MRU.

Supplementary Table 2 Functional annotation of operome involved in transporter mechanisms of MRU.

Supplementary Data All predicted information for functional assignment of MRU operome.
