# Supplementary material for: Functional Prediction and Assignment of Methanobrevibacter ruminantium M1 Operome Using a Combined Bioinformatics Approach
Source: Front Genet. 2020 Dec 16;11:593990. doi: 10.3389/fgene.2020.593990 (PMC7772410; doi:10.3389/fgene.2020.593990)
Supplement: Supplementary Table 1 — Functional annotation of operome involved in diverse metabolic systems of MRU. [file Data_Sheet_1.docx]

**Table S1** Functional annotation of operome involved in diverse metabolic systems of MRU

| Locus tag | Assigned function | EC | Gene | Assignment |
| --- | --- | --- | --- | --- |
| 0035\|0277 | NUDIX hydrolase | 3.6.1.22 | nadM | UCF |
| 0062\|0063 | Archaea-specific DNA helicase | 3.6.4.12 | ashA | UCF |
| 0156\|0041 | E3 SUMO-protein ligase | 6.3.2.- | piaS4 | KF |
| 0202 | Aconitate hydratase | 4.2.1.3 | acnA | UCF |
| [0313](http://www.genome.jp/dbget-bin/www_bget?mru:mru_0313) | Pyrogallol hydroxytransferase | 1.97.1.2 | athL | KF |
| 0381 | Succinylglutamate desuccinylase | 3.5.1.96 | astE | KF |
| 0393 | Adenylate kinase | 2.7.4.3 | adk | UCF |
| [0421](http://www.genome.jp/dbget-bin/www_bget?mru:mru_0421) | Alpha-2,3-sialyltransferase | 2.4.99.4 | SIAT4A | KF |
| 0426\|1311 | tRNA-splicing ligase | 6.5.1.3 | rtcB | UCF |
| 0460 | Dolichol kinase | 2.7.1.108 | dolk | UCF |
| 0476 | Phenylacetate-CoA oxygenase | 1.14.13.149 | paaJ | KF |
| 0480 | Pyruvate kinase | 2.7.1.40 | pykA | UCF |
| 0496\|1242 | ATP pyrophosphatase | 3.6.1.8 | thiI | KF |
| 0596 | 4-Hydroxybenzoate octaprenyltransferase | 2.5.1.39 | ubiA | UCF |
| 0720 | UMP kinase | 2.7.4.22 | pyrH | UCF |
| 0747 | 2-Polyprenylphenol 6- hydroxylase | 1.14.13.- | ubiB2 | UCF |
| [0791](http://www.genome.jp/dbget-bin/www_bget?mru:mru_0791) | Methylated-DNA-[protein]-cysteine S-methyltransferase | 2.1.1.63 | - | UCF |
| 0850 | tRNA (guanine^37^-N^1^)-methyltransferase | 2.1.1.228 | trmD | KF |
| [0947](http://www.genome.jp/dbget-bin/www_bget?mru:mru_0947) | Coenzyme F_420_-0:L-glutamate ligase | 6.3.2.31 | cofE | KF |
| 1013 | 6-Phosphogluconate dehydrogenase | 1.1.1.343 | gntZ | KF |
| 1065 | CDP-glycerol glycerophosphotransferase | 2.7.8.12 | tagF | UCF |
| 1096 | Serine/threonine protein kinase | 2.7.11.1 | bub1 | UCF |
| 1116 | CTP: Molybdenum cofactor cytidylyltransferase | 2.7.7.76 | mocA | KF |
| 1209 | Nicotinate-nucleotide pyrophosphorylase | 2.4.2.19 | nadC | UCF |
| 1280\|1936 | NADPH-dependent FMN reductase | 1.5.1.38 | ssuE | UCF |
| 1290\|1291 | Lincosamide nucleotidyltransferase | 2.7.7.- | inuA | UCF |
| 1330 | Carbohydrate kinase | 2.7.1.4 | pfkB | UCF |
| [1344](http://www.genome.jp/dbget-bin/www_bget?mru:mru_1344) | Lysine carboxypeptidase | 3.4.17.3 | CPN1 | KF |
| 1375\|1884 | Dolichyl-phosphate-mannose-protein mannosyltransferase | 2.4.1.109 | pomT | KF |
| 1418 | dTDP-4-amino-4,6-dideoxygalactose transaminase | 2.6.1.59 | rffA | KF |
| 1442 | Geranylgeranyl reductase | 1.3.1.83 | chlP | UCF |
| 1450 | Energy-converting hydrogenase B subunit O | 1.6.5.3 | ehbO | KF |
| 1462 | Pantothenate synthase | 6.3.2.1 | panC | UCF |
| 1469 | UDP-glucose 4-epimerase | 5.1.3.2 | galE | UCF |
| 1493 | Tryptophanyl-tRNA synthetase | 6.1.1.2 | trpS | UCF |
| [1563](http://www.genome.jp/dbget-bin/www_bget?mru:mru_1563) | Proteasome endopeptidase complex | 3.4.25.1 | psmA | UCF |
| 1589\|1957 | Thiamine monophosphate synthase | 2.5.1.3 | thiE | UCF |
| [1605](http://www.genome.jp/dbget-bin/www_bget?mru:mru_1605) | Nucleotide diphosphatase | 3.6.1.9 | ENPP1_3 | KF |
| 1622 | 4-Carboxymuconolactone decarboxylase | 4.1.1.44 | pcaC | UCF |
| [1631](http://www.genome.jp/dbget-bin/www_bget?mru:mru_1631) | UDP-N-acetylglucosamine 2-epimerase | 5.1.3.14 | wecB | UCF |
| 1693 | Phosphatidate cytidylyltransferase | 2.7.7.41 | cdsA | KF |
| [1696](http://www.genome.jp/dbget-bin/www_bget?mru:mru_1696) | Carbamoyl-phosphate synthase (glutamine-hydrolyzing) | 6.3.5.5 | carB | UCF |
| 1728 | Phosphomevalonate decarboxylase | 4.1.1.99 | pmd | KF |
| 1737 | Saccharopine dehydrogenase (NAD/P, L-lysine-forming) | 1.5.1.7\|1.5.1.8 | lys1 | KF |
| 1749 | Succinate dehydrogenase/fumarate reductase | 1.3.99.1 | sdh | UCF |
| 1867 | Ribosomal-protein-alanine N-acetyltransferase | 2.3.1.128 | rimI | UCF |
| 1831 | Dihydroneopterin aldolase | 4.1.2.25 | folB | UCF |
| 1862 | Cu^+^-exporting ATPase | 3.6.3.54 | copA | KF |
| 1886 | Glycogen phosphorylase | 2.4.1.1 | glgP | KF |
| 1890 | Phosphoribosylaminoimidazole carboxylase | 4.1.1.21 | purE1 | UCF |
| 1894 | Fumarate hydratase | 4.2.1.2 | fumA4 | UCF |
| 1938 | Arsenate reductase | 1.20.4.1 | arsC | KF |
| 2069 | DNA-3-methyladenine glycosylase | 3.2.2.20 | tag | UCF |
| 2146 | Phospho-N-acetylmuramoyl-pentapeptide transferase | 2.7.8.13 | MraY | UCF |
| 2158 | Pyruvate formate-lyase | 1.97.1.4 | pflX | UCF |
| [2172](http://www.genome.jp/dbget-bin/www_bget?mru:mru_2172) | Riboflavin kinase | 2.7.1.161 | ribK | UCF |
| 2180\|2184\|2185 | Acyltransferase | 2.3.1.13 | GLYAT | KF |
| 2194 | 2-Enoyl-CoA Hydratase | 3.4.21.92 | clpP | KF |
| 2196 | Choloylglycine hydrolase | 3.5.1.24 | - | UCF |
| 2219 | Cobalamin biosynthesis protein | 6.3.1.10 | cbiB | UCF |
| 1317 | Diaminohydroxyphosphoribosylaminopyrimidine reductase | 3.5.4.26 | RibD | KF |
| 1323 | Tungsten formylmethanofuran dehydrogenase subunit E | 1.2.99.5 | FwdE | UCF |
| 1857 | Cobyrinic acid a,c-diamide synthase | 6.3.5.9/6.3.5.11 | CbiA2 | UCF |
| 1997 | CRISPR/Cas system-associated protein 4 | 3.1.12.1 | Cas4 | KF |

KF: Protein with known function; UCF: Protein with uncovered function

**Table S2** Functional annotation of operome involved in transporter mechanisms of MRU

| Locus tag | Assigned function | TC | Gene |
| --- | --- | --- | --- |
| 0283 | Vitamin Uptake Transporter | 2.A.88 |  |
| 0361\|  0372\| 0717 | Sulfolactate (sulfite/organosulfonate) exporter | 2.A.102.2.1 | *tauE/safE* |
| 0428 | Putative membrane protein | 9.B.98.1.3 | *-* |
| 0521 | TctA homologue | 2.A.80.2.1 | *tctA* |
| 0542 | Magnesium ion transporter | 1.A.26 | *mgtE* |
| 0607 | Mn^+^ ion transporter | 2.A.4 | *-* |
| 0738\|0739\|  0742\|0761\|  1227 | Translocon sheath protein | 1.C.36.5.1 | *sseB* |
| 1597 | Preprotein translocase | 3.A.5 | *secY* |
| 1984 | Putative permease | 2.A.86.1.1 | *perM* |
| 2130 | Na^+^/H^+^ antiporter protein | 2.A.33 | *NhaA* |
